# Malaria Infected Mosquitoes Express Enhanced Attraction to Human Odor

**DOI:** 10.1371/journal.pone.0063602

**Published:** 2013-05-15

**Authors:** Renate C. Smallegange, Geert-Jan van Gemert, Marga van de Vegte-Bolmer, Salvador Gezan, Willem Takken, Robert W. Sauerwein, James G. Logan

**Affiliations:** 1 Laboratory of Entomology, Wageningen University and Research Center, EH Wageningen, The Netherlands; 2 Department of Disease Control, London School of Hygiene and Tropical Medicine, Keppel Street, London, United Kingdom; 3 Department of Medical Microbiology, Radboud University Nijmegen Medical Centre, HB Nijmegen, The Netherlands; 4 Department of Statistics, Institute of Food and Agricultural Sciences, University of Florida, Gainesville, Florida, United States of America; Johns Hopkins University, Bloomberg School of Public Health, United States of America

## Abstract

There is much evidence that some pathogens manipulate the behaviour of their mosquito hosts to enhance pathogen transmission. However, it is unknown whether this phenomenon exists in the interaction of *Anopheles gambiae sensu stricto* with the malaria parasite, *Plasmodium falciparum* - one of the most important interactions in the context of humanity, with malaria causing over 200 million human cases and over 770 thousand deaths each year. Here we demonstrate, for the first time, that infection with *P. falciparum* causes alterations in behavioural responses to host-derived olfactory stimuli in host-seeking female *An. gambiae* s.s. mosquitoes. In behavioural experiments we showed that *P. falciparum*-infected *An. gambiae* mosquitoes were significantly more attracted to human odors than uninfected mosquitoes. Both *P. falciparum-*infected and uninfected mosquitoes landed significantly more on a substrate emanating human skin odor compared to a clean substrate. However, significantly more infected mosquitoes landed and probed on a substrate emanating human skin odor than uninfected mosquitoes. This is the first demonstration of a change of *An. gambiae* behaviour in response to olfactory stimuli caused by infection with *P. falciparum*. The results of our study provide vital information that could be used to provide better predictions of how malaria is transmitted from human being to human being by *An. gambiae s.s.* females. Additionally, it highlights the urgent need to investigate this interaction further to determine the olfactory mechanisms that underlie the differential behavioural responses. In doing so, new attractive compounds could be identified which could be used to develop improved mosquito traps for surveillance or trapping programmes that may even specifically target *P. falciparum*-infected *An. gambiae s.s.* females.

## Introduction

There is evidence that some parasites manipulate the behavior of their vectors to enhance pathogen transmission [1]. For example, the malaria mosquito *Anopheles gambiae*, infected with transmissible sporozoite stages of the human malaria parasite *Plasmodium falciparum*, takes larger and more frequent blood meals than uninfected mosquitoes or those infected with non-transmissible oocyst forms [2]. This parasite-mediated manipulation of behavior in *An. gambiae* is likely to facilitate parasite transmission from human to mosquito.

It would be even more advantageous for the parasite if its vector is more responsive to host odors, as this is the dominant cue used to find a blood meal. Indeed, Rossignol *et al.* [3] found that a higher percentage of *Aedes aegypti* mosquitoes respond to guinea pig odor in an olfactometer when infected with the avian malaria parasite *P. gallinaceum* compared to uninfected females, demonstrating that parasites affect the host-seeking behavior of mosquitoes. However, it is unknown whether this phenomenon exists in other, biologically more important, systems.

We investigated whether parasite manipulation exists in the *An. gambiae sensu stricto* - *P. falciparum* parasite interaction, responsible for one of the most important human infectious diseases. We hypothesized that infection with *P. falciparum* causes alterations in olfactory-mediated behavioral responses to host-derived stimuli in host-seeking *An. gambiae* mosquitoes. Indeed, our study demonstrates that females of *An. gambiae* infected with sporozoites of *P. falciparum*, are significantly more attracted in a laboratory setting to human odors than uninfected mosquitoes.

## Results and Discussion

We collected human odors using a nylon matrix for the attraction of host-seeking *An. gambiae* mosquitoes. The matrix, and a control matrix which did not contain human odor, was presented to the mosquitoes in a cage olfactometer to measure the landing responses. At the time of the experiment, mosquitoes were either uninfected or infected with sporozoite-stage *P. falciparum.*

**Figure 1:**
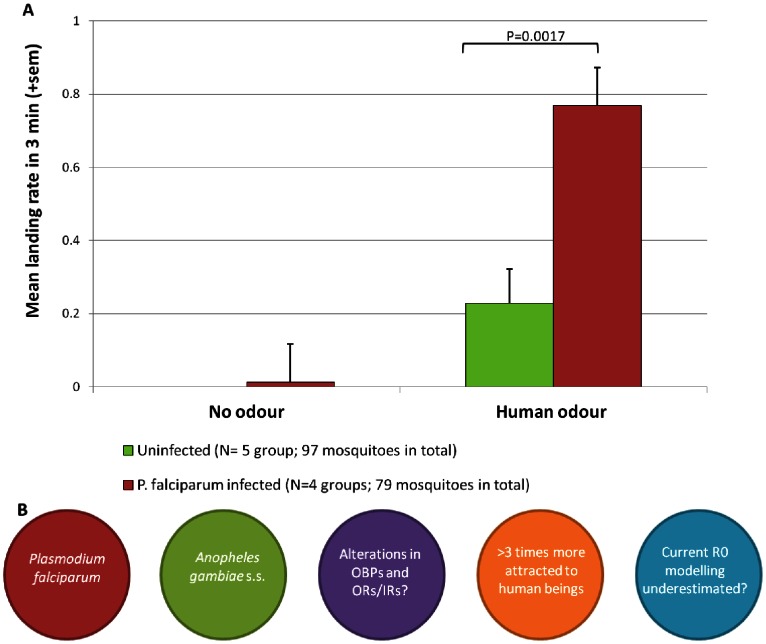
Attraction of malaria infected mosquitoes to human odor. (**A**) Total number of landings by uninfected (green bars) and *P. falciparum* infected (red bars) *An. gambiae s.s.* females in response to no odor (left bars) or human odor (right bars). Error bars represent the standard error of the mean. (**B**) Simplified overview of our hypothesis on the effect of *P. falciparum* infection of *An. gambiae s.s.* on human malaria risk via alterations in the olfactory system of the vector (OBPs: odorant-binding proteins, ORs/IRs: olfactory and ionotropic receptors; R0: the basic reproductive number).

Statistical analysis revealed a significant effect on the mosquito landing rate response of both the odor source used and the presence of *P. falciparum*, and a significant interaction between the two factors (*P*<0.001, *P* = 0.014, and *P = *0.018, respectively). As expected, both infected and uninfected mosquitoes showed a low rate of landing on the matrix without human odor. However, infected mosquitoes responded significantly more to the matrix on which human odor was collected than uninfected (Figure 1A). Malaria-infected mosquitoes performed significantly more landings and probing attempts in response to human odor than did uninfected mosquitoes (*P* = 0.0017). These results suggest that malaria-infectious females are more attracted to human odors than uninfected mosquitoes (Figure 1A). This is the first indication of a change in *An. gambiae s.s.* behavior in response to human odor, caused by infection with *P. falciparum*. So far, most studies of *An. gambiae* mosquito behavior have been conducted with uninfected mosquitoes, but our data demonstrate that such results may not be representative of infected mosquitoes. Mathematical models incorporating malaria transmission are considered important tools for development of malaria eradication strategies [4]. A number of mathematical models take into consideration various factors that affect R_0_, the basic reproductive number, which describes the number of secondary human infections that arise from a primary infection [5], but do not address the influence of parasites on vector-host interactions.

Naturally, it would be most advantageous for a parasite if its vector is more responsive to host odors once the parasite has developed into the transmissible stage, and not at an earlier stage [1]. To study this aspect properly, the host-seeking response of uninfected vectors and vectors infected with immature and mature parasite stages should be compared. Indeed, Anderson et al. [6] showed that changes in *An. stephensi* females feeding behaviour were dependent on the developmental stage of the parasite *P. yoelii nigeriensis* (a malaria parasite of rodents). Therefore, for the primary study, described in this paper, we studied the effect of the stage of *P. falciparum* transmissible to human beings. Because these initial results supported our hypothesis, we have already initiated robust behavioural studies to ensure that our preliminary findings are repeatable and determine in more detail whether the effect on the host-seeking behaviour of *An. gambiae s.s.* depends on the lifecycle stage of *P. falciparum* and in this study we will test odors from multiple human subjects.

Lefèvre *et al*. [7] showed that infection with the rodent malaria parasite, *P. berghei*, altered levels of 12 protein spots in the head of *An. gambiae* infected with sporozoites, including synapse-associated proteins, likely affecting the olfactory system. Therefore, it is likely that the mechanism underlying the behavioral difference in infected mosquitoes lies within the olfactory system, possibly mediated by alteration of odorant-binding proteins (OBPs) or olfactory and ionotropic receptors (ORs and IRs; Figure 1B) tuned to host-derived semiochemicals [8]. Further studies on the identification of new attractants for improved mosquito surveillance or trapping programs specifically targeting *P. falciparum*-infected *An. gambiae s.s.* females may provide powerful tools for the global agenda of malaria eradication.

## Materials and Methods

### Mosquitoes

*Anopheles gambiae sensu stricto* mosquitoes (Ngousso strain from Cameroon) were reared according standard procedures at the insectaries of Radboud University Nijmegen (The Netherlands). 9-Days-old female mosquitoes had been given the opportunity to blood feed on human blood, either uninfected or infected with *Plasmodium falciparum* parasites, by using a membrane glass feeding system [9]. Unfed females were removed from the cages. All groups of females were given an opportunity to oviposit 3 days post blood feeding.

Infected female mosquitoes were obtained by feeding on gametocytes of the chloroquine-sensitive NF54 strain of *P. falciparum*, as described previously [9]. Per cage, 10 blood-engorged mosquitoes were dissected at day 7 post blood feeding showing that 95% of the females were infected with on average 10 oocysts per mosquito (N = 20).

All mosquitoes received another human blood meal nine days after the previous blood meal. Twenty one days after the first blood meal, cages (30×30×30 cm) were prepared, some containing 20 uninfected females, and others containing 20 infectious females. On day 22, after the bioassay had been conducted, salivary glands of 10 females per cage (N = 40) were dissected showing that *P. falciparum* sporozoites had migrated into 84% of the glands. Twenty mosquitoes were dissected to determine the average of 18.125 sporozoites per mosquito. The cages were randomly numbered and only the technician who prepared the mosquitoes (not the experimenter) was aware of their infectivity status.

### Bioassay

Considering the high degree of anthropophily of *An. gambiae s.s.* females and the practical and effective use of human foot odor *in vitro*, human foot odor was collected on a nylon matrix as described previously [10] (20 Den panty sock, HEMA, The Netherlands, worn during 20 hours prior to the day on which the bioassay was performed by a male volunteer of whom the relative attractiveness to *An. gambiae s.s.* compared to 47 other men is known [11]). A clean matrix was used as the control. Prior and between experiments, matrixes were kept, individually, in clean glass jars.

The bioassay was conducted between 8 and 11 am, in a BSL-3 climate cell (26±1°C, 80±10% R.H.). During the bioassay the room was dark except for a light bulb of 15 Watt (Osram, France) pointed at the wall behind the cage being observed. For each cage the number of landings in the area directly underneath the odor-treated matrix or the clean matrix was recorded over 3 minutes.

### Statistics

After completion of the bioassay, the infectivity status corresponding with the numbers on the cages were revealed to the scientist who subsequently analyzed the results. We used the rate of landing (i.e. the number of landings per mosquito), rather than total number of landings as this takes into account the fact that a particular mosquito may land more than once. The rate of landing was calculated by dividing the total number of landings by the total number of mosquitoes used in the bioassay to give the mean landing rate per mosquito. This response was then analysed using a two-way ANOVA to compare the effect of odor and *Plasmodium* infection, and their interaction (GenStat version 15.2.0.8821) The Least Significant Differences (LSDs) were used to calculate P-values for the significant difference between the treatments.

### Ethics

The author/experimenter performed odour collection on herself by wearing nylon stockings.

Mosquitoes were bloodfed using membrane and a Hemotek blood feeding system. The blood was obtained from Sanguin, Nijmegen, The Netherlands and the blood donors signed an informed consent. No human volunteers were used for feeding the mosquitoes. The rearing of mosquitoes using this procedure is standard practice in many laboratories. The blood is not used for experimental purposes. No volunteers were recruited, therefore, ethical approval was not necessary.
